# Latent class analysis of sexual health markers among men and women participating in a British probability sample survey

**DOI:** 10.1186/s12889-019-7959-7

**Published:** 2020-01-09

**Authors:** Alison Parkes, Michael Waltenberger, Catherine Mercer, Anne Johnson, Kaye Wellings, Kirstin Mitchell

**Affiliations:** 1 MRC/CSO Social and Public Health Sciences Unit, University of Glasgow, Top floor, 200 Renfield St, Glasgow, UK; 2 UCL Institute for Global Health, University College London, Mortimer Market Centre, Off Capper Street, London, UK; 3 Faculty of Public Health and Policy, London School of Hygiene and Tropical Medicine, Tavistock Place, London, UK

**Keywords:** Sexual health, Sexual wellbeing, Sexual function, Unplanned pregnancy, Sexually transmitted infection, Sexual coercion

## Abstract

**Background:**

Despite known associations between different aspects of sexual health, it is not clear how patterning of adverse sexual health varies across the general population. A better understanding should contribute towards more effective problem identification, prevention and treatment. We sought to identify different clusters of sexual health markers in a general population, along with their socio-demographic, health and lifestyle correlates.

**Methods:**

Data came from men (*N* = 5113) and women (*N* = 7019) aged 16–74 who reported partnered sexual activity in the past year in Britain’s third National Survey of Sexual Attitudes and Lifestyles, undertaken in 2010–2012. Latent class analysis used 18 self-reported variables relating to adverse sexual health outcomes (STI and unplanned pregnancy, non-volitional sex, and sexual function problems). Correlates included socio-demographics, early debut, alcohol/drug use, depression, and satisfaction/distress with sex life.

**Results:**

Four classes were found for men (labelled Good Sexual Health 83%, Wary Risk-takers 4%, Unwary Risk-takers 4%, Sexual Function Problems 9%); six for women (Good Sexual Health 52%, Wary Risk-takers 2%, Unwary Risk-takers 7%, Low Interest 29%, Sexual Function Problems 7%, Highly Vulnerable 2%). Regardless of gender, Unwary Risk-takers reported lower STI/HIV risk perception and more condomless sex than Wary Risk-takers, but both were more likely to report STI diagnosis than Good Sexual Health classes. Highly Vulnerable women reported abortion, STIs and functional problems, and more sexual coercion than other women. Distinct socio-demographic profiles differentiated higher-risk classes from Good Sexual Health classes, with depression, alcohol/drug use, and early sexual debut widely-shared correlates of higher-risk classes. Females in higher-risk classes, and men with functional problems, evaluated their sex lives more negatively than those with Good Sexual Health.

**Conclusions:**

A greater prevalence and diversity of poor sexual health appears to exist among women than men in Britain, with more consistent effects on women’s subjective sexual well-being. Shared health and lifestyle characteristics of higher-risk groups suggest widespread benefits of upstream interventions. Several groups could benefit from tailored interventions: men and women who underestimate their STI/HIV risk exposure, women distressed by low interest in sex, and women experiencing multiple adverse outcomes. Distinctive socio-demographic profiles should assist with identification and targeting.

## Background

The World Health Organization (WHO)’s holistic conceptualization of sexual health refers to pleasurable and safe sexual experiences, free from disease, dysfunction and coercion, recognising the importance of psychosocial as well as physiological, dimensions [1]. Underlining this holistic viewpoint, an extensive literature demonstrates associations between various domains of poor sexual health, relating to sexually transmitted infections (STIs) and unwanted pregnancy risk, sexual function problems, and sexual coercion [2–8]. Nonetheless, it is important to note that correlations between different domains are often modest; and that domains have distinct, as well as common, socio-demographic, health and lifestyle predictors. For instance, a British national probability survey found that socio-economic disadvantage and substance use were correlates of sexual coercion, STIs, and unwanted pregnancy, but not low sexual function [2, 6, 9–11]. The survey also found that depression was more strongly correlated with sexual coercion and function problems than with STI or unplanned pregnancy risk [2, 6, 10, 12]. This suggests population heterogeneity in the distribution of adverse sexual health outcomes across domains. There are also indications of within-domain heterogeneity, because different within-domain markers of poor sexual health are not always consistently aligned and may have different predictors [13–17]. Population heterogeneity in the distribution of adverse sexual health markers, although currently not well understood, has implications for the targeting and delivery of sexual health programmes. A holistic perspective envisages likely benefits of integrated sexual health intervention streams, and of co-ordinated approaches to sexual and reproductive health [18, 19]; however, it is acknowledged that targeted sexual health interventions may be most appropriate in addressing specific influences and motivations affecting particular groups [20]. A better understanding of how different aspects of sexual health commonly cluster in the general population should contribute towards more effective problem identification, and towards ascertaining the optimum balance between universal vs. targeted prevention and treatment.

This paper seeks to describe how multiple different aspects of sexual health co-occur in a general population, together with the socio-demographic, health, and lifestyle correlates of different clusters. In so doing, we aim to address commonly-overlooked issues such as diversity of sexual health needs across the lifecourse; and the relevance of psychosocial as well as physiological factors for subjective sexual well-being [21]. While current evidence supports a holistic viewpoint in suggesting various aspects of poor sexual health all negatively impact sexual well-being [22–26], there are gaps in knowledge. It is unclear whether engaging in behaviour that carries a future STI risk impacts sexual well-being to the same extent as STI diagnosis. It is also unclear whether poor sexual health affects men and women’s perceived sexual well-being equally [25, 27, 28]. Further research would help clarify where improving sexual well-being is likely to be an important element of sexual health interventions directed at different population groups.

Traditional analysis methods do not allow us to assess multiple dimensions of sexual health simultaneously, to uncover heterogeneity in their co-occurrence. In this study, we use latent class analysis to identify different clusters of sexual health markers in a nationally representative British sample. Compared with standard cluster analysis techniques such as k-means or hierarchical cluster analysis, latent class analysis has several advantages: these include a model-based approach classifying study respondents into the appropriate cluster on the basis of estimated membership probabilities, greater use of formal criteria to decide on the final model, and greater flexibility in accommodating variables measured on different scales [29]. Latent class analysis can help address methodological challenges such as high Type I error rates and low statistical power that commonly arise in traditional subgroup analyses, where each group has been defined a priori based on certain characteristics [30]. This technique has previously been used within specialised areas of sexual health, for example to investigate different patterns of risk-taking behaviours or victimization experiences within high-risk groups [31–33]. To our knowledge, latent class analysis has not been applied to examine within- and across-domain clustering across a broad spectrum of sexual health outcomes, in the general population.

We ask the following research questions:

1. How do different markers of sexual health cluster among sexually active individuals, and how does clustering vary by gender?
2. How do socio-demographic, lifestyle and health factors characterise membership of different sexual health clusters?
3. How is membership of different sexual health clusters associated with subjective sexual wellbeing, defined in terms of satisfaction/distress with one’s sex life?

## Methods

This study used data from the third wave of the British National Survey of Sexual Attitudes and Lifestyles (Natsal-3), a stratified probability sample survey of 15,162 men and women aged 16–74 years in Britain (England, Scotland, and Wales), interviewed in 2010–12. The estimated response rate was 57.7%, while the cooperation rate was estimated at 65.8% of all eligible addresses contacted [34]. In-home computer-assisted interviews were conducted using a combination of face-to-face questions and a self-complete module for sensitive items. Details of the survey methods and questionnaire are available on the study website, see http://www.natsal.ac.uk/natsal-3.aspx and elsewhere [35]. The Natsal-3 study was approved by the Oxfordshire Research Ethics Committee A (10/H0604/27). Respondents provided oral informed consent for interviews.

### Measures

All measures used self-reported information (details of measures and sample information are provided in Table 1).

**Table 1:** Measures of sexual health, sociodemographic, health and lifestyle factors

Measure	Detailed information	Subgroups (if applicable)	Men (%)	Women (%)
STI/Unplanned pregnancy				
Unplanned pregnancy	Based on the London Measure of Unplanned Pregnancy [36], a score of six items asking women reporting a pregnancy in the past year about contraceptive use, timing of motherhood, intention to become pregnant, desire for a baby, discussion with a partner, and preconceptual preparations. Scores range from 0 to 12, with unplanned pregnancy defined as a score of 3 or less.		–	1.2
Abortion	Based on questions asked of women about experience of abortion, and age at last abortion. Responses restricted to those reporting an abortion in the last 5 years.		–	3.1
STI diagnosis	“Have you ever been told by a doctor or other healthcare professional that you had any of the following?” Sexually transmitted infections asked about included chlamydia, gonorrhoea, genital warts (venereal warts), syphilis, Trichomonas vaginalis (Trich, TV), Herpes (genital herpes), NSU (Non Specific Urethritis), NGU (Non Gonococcal Urethritis). Responses were restricted to diagnoses made in the last 5 years.		4.2	4.0
Perceived STI risk	“There are different opinions about how many people are at risk of becoming infected with HIV, the virus that causes AIDS, but we would like to know what you think about the risks to you, personally with your present sexual lifestyle?” Responses 1 “greatly at risk” and 2 “quite a lot” were combined and contrasted with 3 “not very much” and 4 “not at all at risk”.		4.1	2.7
Perceived HIV risk	“People are also at risk of getting other sexually transmitted infections. What do you think about the risks to you, personally, with your present lifestyle of getting a sexually transmitted infection?” Responses 1 “greatly at risk” and 2 “quite a lot” were combined and contrasted with 3 “not very much” and 4 “not at all at risk”.		3.5	2.6
Condomless sex (2+ partners)	At least one episode of non-use of condoms, occurring with two or more partners in the past year		6.7	5.1
Condomless sex (first sex with new partner)	Non-use of condoms at first sex with most recent new partner in the past year		15.2	13.4
Sexual coercion				
Non-volitional sex	“Since the age of 13, has anyone actually made you have sex with them, against your will?” (yes/no)		1.4	10.3
First sexual partner more willing	“Would you say that you were both equally willing to have intercourse that first time, or was one of you more willing than the other?” Response 3 “(He/She) was more willing” was contrasted with responses 1 “Both equally willing” and 2 “I was more willing”. Item applied to first sex with an opposite-sex partner, after the age of 13.		4.4	16.2
Sexual Function				
Lacked interest in sex	Problem experienced for at least 3 months during the past year (yes/no)		15.0	34.1
Lacked enjoyment in sex	Problem experienced for at least 3 months during the past year (yes/no)		4.8	12.1
Anxious during sex	Problem experienced for at least 3 months during the past year (yes/no)		5.4	5.2
Physical pain from sex	Problem experienced for at least 3 months during the past year (yes/no)		1.8	7.4
Lack of arousal	Problem experienced for at least 3 months during the past year (yes/no)		3.1	8.2
Trouble experiencing orgasm	Problem experienced for at least 3 months during the past year (yes/no)		9.2	16.3
Premature orgasm	Problem experienced for at least 3 months during the past year (yes/no)		14.9	2.3
Erectile difficulties/dry vagina	Problem experienced for at least 3 months during the past year (yes/no)		12.1	12.2
Avoided sex	Agreement with “I have avoided sex because of sexual difficulties, either my own or those of my partner”. Response options 1 “agree strongly” and 2 “agree” were combined and contrasted with 3 “neither agree nor disagree”, 4 “disagree”, 5 “disagree strongly”.		11.0	13.5
Satisfaction/Distress with sex life	Average agreement with statements “I feel satisfied with my sex life”, and “I feel distressed or worried about my sex life”. Responses used a 5-point scale from 1 “agree strongly” to 5 “disagree strongly”. The second item was reverse-scored, so that a high average score denoted less satisfaction/more distress. The top decile (approximately, due to the presence of tied scores) was defined as “low satisfaction/high distress with sex life “and contrasted with the remainder.		11.6	11.2
Socio-demographic, health and lifestyle information				
Age (years)	Age at interview	16–24	15.2	15.6
		25–44	41.3	43.2
		45–54	19.9	20.6
		55–74	23.7	20.6
Ethnicity	White British, White Irish, Any other White background	white	87.9	88.3
	Mixed, Asian, Asian British, Black, British Black, Chinese, Other	minority	12.1	11.7
Low socio-economic status	Markers obtained at the time of interview comprised lowest household income quintile, no educational qualifications, unemployed; highest area deprivation quintile according to the Index of Multiple Deprivation (Payne & Abel, 2012)	zero markers	48.6	42.9
		1 or 2 markers	39.6	42.8
		3 to 5 markers	11.8	14.3
Alcohol/drug use	Defined as either (1) frequent binge drinking (at least once a week) based on current drinking practices; and/or (2) recent non-prescribed drug use (in the past 4 weeks). An episode of binge drinking was defined as consumption of more than six (for women) or eight (for men) units of alcohol on any one occasion. Drugs asked about included cannabis, amphetamines, cocaine, crack cocaine, ecstasy, heroin, lysergic acid diethylamide (LSD), crystal methamphetamine and amyl nitrates.		23.5	17.8
Depression	Based on the patient health questionnaire (PHQ-2) [37], comprising two screening questions (‘During the past 2 weeks have you often been bothered by feeling down, depressed, or hopeless?’, and ‘During the past 2 weeks have you often been bothered by little interest or pleasure in doing things?’) to assess depressive symptoms in the past 2 weeks (each scored 0–3). Respondents were deemed to have depressive symptoms if they had a total score of three or more, a cut-off that has been previously validated.		8.6	10.7
Partnership status	Status at interview	Married/ cohabiting	72.1	73.2
		Regular partner	12.4	13.3
		No regular partner	15.5	13.5
Sexual identity	Responses to “Which of the options on this card best describes how you think of yourself?” with options: (1) Heterosexual/straight, (2) Gay/Lesbian, (3) Bisexual, (4) Other.	Heterosexual/ straight	97.2	97.1
		Gay/lesbian/ bisexual/other	2.8	2.9
Age < 16 years at first sex	“How old were you when you first had sexual intercourse with someone of the opposite sex?” Responses include estimates for those unsure of the exact age. Those under 16 years (“early sexual debut”) were contrasted with the remainder.		28.1	20.2

Percentages allow for the complex survey design

#### Sexual health markers

In order to capture the multi-faceted nature of sexual health risk, we selected markers in three principle domains alluded to in the WHO conceptualization [1]: risk of sexually transmitted infection and unplanned pregnancy, sexual function problems, and sexual coercion. We aimed to describe current health needs across all population groups, regardless of sexual history, by using markers of recent sexual health. Most measures were therefore confined to experiences in the year prior to interview. A few exceptions related to measures where population prevalence in the year prior to interview was low (STI diagnosis, abortion, unplanned pregnancy and sexual coercion, all < 1%). For these measures, a longer time frame was used to increase statistical power.

#### STI and unplanned pregnancy risk

Measures of STI diagnosis and (women-only) abortions and unplanned pregnancy were included. As one-year population prevalence of STI diagnosis and abortion were low, we selected markers covering a 5-year period prior to interview. However, the London Measure of Unplanned Pregnancy [36, 38] was only available for women aged 16–44 reporting a pregnancy in the past year. Supplementary measures of behavioural and attitudinal antecedents were used as an additional guide to more recent STI/unplanned pregnancy risk exposure. Two behavioural measures of condomless sex included: (i) non-use of condoms at least once when the respondent reported two or more partners in the past year, and (ii) non-use of condoms at first sex with a recent new partner in the past year. Two attitudinal measures covered perceived current risk of (i) HIV (human immunodeficiency virus) and (ii) other STIs.

#### Sexual coercion

To maximise statistical power, we selected two markers relating to lifetime experience and at first sex. The first was a measure of completed non-volitional sex since the age of 13, and the second was based on the respondent’s first sex (ever), when the partner was reported as more willing to have sex than the respondent.

#### Sexual function problems

Included measures of nine difficulties experienced during the past year, lasting three months or more: lacked interest in sex, lacked enjoyment during sex, felt anxious about sex life, painful sex, lack of arousal during sex, trouble experiencing orgasm, premature orgasm, erectile dysfunction (men)/uncomfortably dry vagina (women). An additional marker was reporting avoiding sex because of sexual difficulties.

#### Socio-demographic, health and lifestyle correlates

The following factors were examined as potential correlates of latent classes of sexual health: age [16–74], relationship status categorised as one of three groups (married/cohabiting, regular non-cohabiting partner, no regular partner), ethnicity (white vs. ethnic minority), low socio-economic status, SES (a broadly-based score of five markers to increase reliability and ensure applicability across the lifecourse: lowest household income quintile, no educational qualifications, unemployed; highest area deprivation quintile according to the Index of Multiple Deprivation [39], rented tenure, with scores divided into three groups: zero, 1–2 markers, 3 to 5 markers), sexual identity (heterosexual/straight vs gay/lesbian/bisexual/other); alcohol/drug use (drinking in excess of the recommended weekly limit and/or use of non-prescribed drugs in past year); depression measured using the two-item patient Health Questionnaire (PHQ-2), with a cut-off score of three or more [37], early sexual debut (< 16 years, 16 or older).

#### Satisfaction/distress with one’s sex life

In line with others, we based our measure of subjective sexual well-being on satisfaction and distress [40]. We used the mean score of two items (alphas men .71, women .72) concerning agreement with statements “I feel satisfied with my sex life” and **“**I feel distressed or worried about my sex life” (reverse-scored). We applied a cut-off to contrast the bottom 10 % of scores (here defined as “low satisfaction/high distress with sex life”) with the rest. While this cut-off has no clinical significance, on average those with a low score were likely to express disagreement with being satisfied, and agreement with being distressed.

### Analytic strategy

Mixture modelling was used to identify different latent classes of sexual health, among all sexually-active respondents, defined as those reporting sexual activity involving genital contact with 1+ partner(s) in the year prior to interview. Men (*N* = 5113) and women (*N* = 7019) were modelled separately owing to differences in the experience and reporting of sexual behaviours [41] and the sexual scripts that shape these behaviours [42]. Modelling was performed using Mplus version 8 [43], allowing for the complex survey design, with missing outcome data handled using Full Information Maximum Likelihood (FIML). (For an illustrative example of this technique, see https://stats.idre.ucla.edu/mplus/seminars/mplus-class-notes/lca/.) Various model fit statistics were used to help identify the optimum number of classes, together with considerations of the smallest class size and posterior probabilities of class membership [44]. Smaller Akaike Information Criteria (AIC) and Bayesian Information Criteria (BIC) values are preferable, while Entropy values should be close to 1. The Lo, Mendell and Rubin Likelihood Ratio Test (LMR) test indicated whether a model has a better fit than the model with one fewer class (the complex survey design did not permit use of the Bootstrap Likelihood Ratio Test (BLRT) [45].

After identifying the optimum number of latent sexual health classes for men and women, we named each class according to marker(s) with highest estimated probabilities. Next, we sought to ascertain the different socio-demographic, health and lifestyle profiles of the various classes obtained. Multinomial regression models of class membership on socio-demographic, health and lifestyle correlates used an integrated procedure in Mplus (R3STEP), which allows for classification uncertainty following mixture modelling [46]. Correlates were considered in two stages: (a) socio-demographic factors mutually adjusted for one another; followed by (b) alcohol/drug use, depression and early sexual debut each modelled separately, adjusting for socio-demographic information. Missing correlate information was generally at low levels (on average for men 1.7%, and for women 1.5%). Nonetheless, a complete case analysis would have resulted in a loss of 6.3% of cases for men and 6.0% for women, with under-representation of older, minority ethnic, and low socio-economic status respondents. Unlike missing outcome information, missing predictor information could not be handled using FIML. To reduce bias, analysis of latent class correlates was performed on 20 data sets imputed using the Mplus multiple imputation facility [47], allowing for complex survey design as before.

Lastly, we explored latent class membership as a predictor of low satisfaction/high distress with one’s sex life. A regression model of low satisfaction/high distress on latent class used an integrated procedure in Mplus (DCATEGORICAL), which allows for classification uncertainty [48]. In this part of the analysis, software constraints did not permit the use of complex survey features.

## Results

Latent class analysis of sexual health markers explored models with varying numbers of classes, with model fit statistics shown in Table 2.

**Table 2:** Model fit statistics for different numbers of sexual health groups identified using latent class analysis

	Number of classes	AIC	BIC	Entropy	Smallest class (%)	lowest class probability	LMR *p*-value
Men	1	38,361	38,465		100.0	1.00	
	2	36,783	36,998	0.70	18.5	0.86	<.001
	3	35,911	36,238	0.75	10.0	0.85	<.001
	**4**	**35,565**	**36,003**	**0.89**	**4.3**	**0.88**	**<.001**
	5	35,408	35,957	0.71	3.7	0.82	0.330
	6	35,352	36,013	0.70	3.6	0.67	0.182
Women	1	68,151	68,275		100.0	1.00	
	2	63,649	63,903	0.78	16.9	0.90	
	3	62,259	62,643	0.78	11.8	0.89	<.001
	4	61,900	62,414	0.85	2.2	0.86	<.001
	5	61,544	62,188	0.79	2.2	0.72	<.001
	**6**	**61,364**	**62,139**	**0.73**	**2.0**	**0.80**	**0.011**
	7	61,212	62,118	0.72	1.9	0.69	0.385
	8	61,107	62,143	0.73	1.9	0.68	0.403

Figures in bold show the fit of the four-class model selected for men, and the six-class model selected for women

*AIC* Akaike Information Criterion, *BIC* Bayesian Information Criterion, *LMR* Lo-Mendell-Rubin likelihood ratio test

For men, a four-class model was selected as the best fit for the data. For women, we selected a six-class model. Although AIC and BIC markers were slightly lower for models with one more class, Lo-Mendell-Rubin tests indicated no significant improvement in fit. For both sexes, selected models had satisfactory entropy (indicating class separation) and high classification accuracy (entropy: men 0.89, women: 0.73; lowest classification probability: men 0.88, women 0.80). Although the sexual health markers used for women included two (unplanned pregnancy and abortion) that were not available for men, we found that exclusion of these markers in order to provide a closer comparison between the sexes did not affect the 6-fold classification found for women (supplementary analyses, available on request).

Among men (Table 3 part a), a large majority (83%) were at low risk and termed the “Good Sexual Health” class. For this class, the average probability of any marker of poor sexual health was .04 (range .00–.13). The three remaining classes were individually termed Wary Risk-takers (4%), Unwary Risk-takers (4%) and Sexual Function Problems (9%), and because of their greater probability of adverse sexual health outcomes were collectively termed “poor sexual health” classes. Wary Risk-takers’ high probability of condomless sex with most recent partner (.44) was accompanied by high STI/HIV risk perception (.63, .87, respectively). In contrast, Unwary Risk-takers were all likely to report condomless sex (1.00, 1.00), but had low STI/HIV risk perception (.06, .04). A range of functional problems characterised the Sexual Function Problems class, each with an average probability of .35 (range .06 to .54). All three poor sexual health classes were more likely to report STI diagnosis in the last 5 years than the Good Sexual Health class, although the probability of STI diagnosis for Wary Risk-takers (.19) was more than double that found for Unwary-Risk-takers and Sexual Function Problems classes (.06 and .08 respectively). Risk of sexual coercion was low in all male classes.

**Table 3:** Latent classes of sexual health among men and women

Men (n = 5113)	Good Sexual Health (*n* = 4112, 83.1%)	Wary Risk-takers (*n* = 270, 3.8%)	Unwary Risk-takers (*n* = 273, 4.3%)		Sexual Function Problems (458, 8.8%)	
Marker	Probability (95% CI)	Probability (95% CI)	Probability (95% CI)		Probability (95% CI)	
STI risk						
STI diagnosis	0.03 (0.02–0.03)	0.19 (0.14–0.25)	0.06 (0.03–0.09)		0.08 (0.05–0.11)	
Perceived STI risk	0.00 (0.00–0.01)	**0.87 (0.74–1.00)**	0.06 (0.02–0.10)		0.02 (0.00–0.04)	
Perceived HIV risk	0.01 (0.01–0.02)	**0.63 (0.53–0.74)**	0.04 (0.01–0.07)		0.01 (0.00–0.02)	
No condom (2+ partners)	0.02 (0.01–0.02)	0.12 (0.05–0.18)	**1.00 (1.00–1.00)**		0.06 (0.03–0.09)	
No condom (new partner)	0.09 (0.08–0.10)	**0.44 (0.35–0.54)**	**1.00 (1.00–1.00)**		0.16 (0.12–0.21)	
Sexual coercion						
Non-volitional sex	0.01 (0.01–0.01)	0.04 (0.00–0.08)	0.02 (0.00–0.03)		0.04 (0.02–0.06)	
First partner more willing	0.04 (0.03–0.05)	0.08 (0.04–0.12)	0.06 (0.02–0.09)		0.06 (0.04–0.09)	
Sexual function problems						
Lacked interest in sex	0.10 (0.09–0.12)	0.14 (0.08–0.21)	0.09 (0.05–0.13)		**0.54 (0.45–0.63)**	
Lacked enjoyment in sex	0.01 (0.01–0.02)	0.09 (0.03–0.14)	0.04 (0.00–0.07)		**0.32 (0.24–0.40)**	
Anxious during sex	0.02 (0.01–0.02)	0.02 (0.00–0.05)	0.08 (0.03–0.12)		**0.35 (0.28–0.43)**	
Physical pain from sex	0.01 (0.01–0.02)	0.03 (0.01–0.06)	0.02 (0.00–0.04)		0.06 (0.03–0.09)	
Lack of arousal	0.00 (0.00–0.01)	0.05 (0.01–0.09)	0.00 (− 0.01–0.01)		**0.25 (0.18–0.32)**	
Trouble experiencing orgasm	0.05 (0.04–0.06)	0.10 (0.04–0.15)	0.07 (0.03–0.10)		**0.43 (0.35–0.52)**	
Premature orgasm	0.13 (0.12–0.14)	0.13 (0.07–0.19)	0.10 (0.06–0.14)		**0.33 (0.27–0.39)**	
Erectile difficulties	0.08 (0.06–0.09)	0.09 (0.05–0.13)	0.14 (0.08–0.19)		**0.49 (0.42–0.55)**	
Avoided sex	0.07 (0.06–0.09)	0.12 (0.07–0.18)	0.12 (0.06–0.18)		**0.38 (0.32–0.44)**	
Women (*n* = 7019)	Good Sexual Health (*n* = 3675, 52.4%)	Wary Risk-takers (*n* = 143, 2.0%)	Unwary Risk-takers (*n* = 482, 6.9%)	Sexual Function Problems (*n* = 535, 7.6%)	Low Interest (*n* = 2046, 29.1%)	Highly Vulnerable (*n* = 138, 2.0%)
	Probability (95% CI)	Probability (95% CI)	Probability (95% CI)	Probability (95% CI)	Probability (95% CI)	Probability (95% CI)
STI/unplanned pregnancy risk						
Unplanned pregnancy	0.01 (0.01–0.02)	0.02 (0.00–0.05)	0.05 (0.02–0.07)	0.01 (0.00–0.01)	0.01 (0.00–0.01)	0.03 (0.00–0.06)
Abortion	0.02 (0.02–0.03)	0.05 (0.00–0.10)	0.10 (0.07–0.14)	0.03 (0.01–0.04)	0.02 (0.01–0.03)	0.18 (0.09–0.26)
STI diagnosis	0.03 (0.02–0.03)	0.16 (0.09–0.23)	0.10 (0.07–0.13)	0.03 (0.01–0.05)	0.03 (0.02–0.04)	**0.27 (0.18–0.36)**
Perceived STI risk	0.00 (0.00–0.00)	**0.97 (0.81–1.13)**	0.04 (0.01–0.08)	0.00 (0.00–0.01)	0.00 (0.00–0.01)	0.18 (0.09–0.28)
Perceived HIV risk	0.01 (0.00–0.01)	**0.71 (0.55–0.88)**	0.04 (0.00–0.07)	0.00 (0.00–0.01)	0.01 (0.00–0.02)	0.13 (0.05–0.21)
No condom (2+ partners)	0.01 (0.00–0.01)	0.13 (0.05–0.21)	**0.52 (0.39–0.64)**	0.00 (− 0.01–0.01)	0.01 (0.00–0.01)	**0.44 (0.30–0.59)**
No condom (new partner)	0.05 (0.03–0.08)	**0.39 (0.24–0.53)**	**0.92 (0.79–1.06)**	0.02 (− 0.01–0.05)	0.06 (0.04–0.09)	**0.74 (0.57–0.90)**
Sexual coercion						
Non-volitional sex	0.05 (0.03–0.07)	0.13 (0.06–0.20)	0.14 (0.09–0.18)	0.18 (0.13–0.23)	0.16 (0.12–0.20)	**0.32 (0.21–0.43)**
First partner more willing	0.11 (0.08–0.14)	**0.26 (0.17–0.36)**	0.20 (0.15–0.25)	0.21 (0.16–0.26)	0.22 (0.18–0.26)	**0.35 (0.24–0.46)**
Sexual function problems						
Lacked interest in sex	0.18 (0.14–0.21)	0.19 (0.11–0.27)	0.20 (0.14–0.26)	**0.94 (0.89–0.98)**	**0.51 (0.42–0.59)**	**0.57 (0.44–0.71)**
Lacked enjoyment in sex	0.01 (−0.01–0.02)	0.06 (0.00–0.11)	0.02 (− 0.01–0.04)	**0.84 (0.74–0.93)**	0.14 (0.08–0.19)	**0.56 (0.41–0.72)**
Anxious during sex	0.00 (0.00–0.01)	0.07 (0.01–0.14)	0.04 (0.01–0.07)	**0.28 (0.22–0.34)**	0.07 (0.04–0.10)	**0.28 (0.18–0.39)**
Physical pain from sex	0.00 (−0.01–0.01)	0.00 (0.00–0.00)	0.04 (0.01–0.06)	**0.34 (0.27–0.42)**	0.13 (0.08–0.18)	**0.25 (0.16–0.34)**
Lack of arousal	0.00 (−0.01–0.01)	0.05 (0.01–0.09)	0.01 (− 0.01–0.03)	**0.67 (0.58–0.77)**	0.07 (0.03–0.10)	**0.47 (0.32–0.61)**
Trouble experiencing orgasm	0.06 (0.04–0.08)	0.10 (0.03–0.17)	0.10 (0.06–0.15)	**0.69 (0.60–0.78)**	0.19 (0.14–0.24)	**0.75 (0.64–0.87)**
Premature orgasm	0.01 (0.01–0.02)	0.01 (− 0.01–0.02)	0.03 (0.01–0.05)	0.04 (0.02–0.07)	0.03 (0.02–0.04)	0.09 (0.02–0.17)
Uncomfortably dry vagina	0.04 (0.03–0.06)	0.02 (0.00–0.05)	0.03 (0.00–0.06)	**0.42 (0.35–0.49)**	0.20 (0.14–0.27)	**0.37 (0.26–0.48)**
Avoided sex	0.00 (−0.02–0.02)	0.10 (0.04–0.16)	0.08 (0.04–0.12)	**0.48 (0.41–0.56)**	**0.28 (0.20–0.36)**	**0.35 (0.23–0.47)**

Figures show estimated probability and associated confidence intervals for markers of poor sexual health. Figures in bold show probabilities ≥ .25, an arbitrary threshold selected to highlight differences between classes

Among women (Table 3 part b), there was a smaller majority in the Good Sexual Health class than observed for men (52% vs 83%), with an average probability of .03 (range .00 to .18) for any marker of poor sexual health. Among women with greater probability of poor sexual health, there were three classes termed Wary Risk-taking (2%), Unwary Risk-taking (7%), Sexual Function Problems (8%) that were similar to the corresponding male classes. Wary Risk-takers’ high risk of condomless sex with most recent partner (.39) was accompanied by high STI/HIV risk perception (.71, .97, respectively). Unwary Risk-takers were likely to report condomless sex (.52, .92) but all perceived themselves as having low STI/HIV risk (.04, .04, respectively). Female Unwary Risk-takers were more likely than the Good Sexual Health class to report an unplanned pregnancy in the past year, or an abortion in the last 5 years. Both female risk-taking classes had a similar elevated risk of STI diagnosis. A range of functional problems characterised the Sexual Function Problems class, each with an average probability of .52 (range .04 to .94). The female Sexual Function Problems class did not differ from the Good Sexual Health class in terms of unplanned pregnancy, abortion or STI diagnosis.

Two additional “poor sexual health” classes were found among women. A large “Low Interest” class (29%) was characterised by lack of interest in sex (probability .51) and avoidance of sex (.28), despite the study population being defined as sexually active in the last year. A small “Highly Vulnerable” class (2%) were likely to report condomless sex (.44, .74), low STI/HIV risk perception (.13, 18, respectively) and a range of sexual problems (average probability .41). This class were more likely to report an abortion (.18) than all other female classes with the exception of Unwary Risk-takers; and most likely to report STI diagnosis (.27). All female poor sexual health classes were more likely to report completed non-volitional sex since age 13 and a more willing partner at first sex, when compared to the Good Sexual Health class. The highest probabilities of coercive sex (.32, .35) were found for the Highly Vulnerable class.

Socio-demographic factors were explored as correlates of sexual health latent class membership; see Tables 4 (Men) and 5 (Women), stage (a). Largely distinctive socio-demographic profiles of the various poor sexual health classes were found, accompanied by notable gender similarities and differences. Compared to those in Good Sexual Health, men and women reporting STI/unplanned pregnancy risk (including Highly Vulnerable women) were characterized by a low SES score, but low SES did not differentiate those experiencing sexual function problems (including Low Interest women). Men in all poor sexual health classes were less likely to be in a married or co-habiting relationship, but this applied only to female classes reporting STI/unplanned pregnancy risk. In contrast, women in the Sexual Function Problem class were more likely to be married or cohabiting than those in good health; while the female Low Interest class was primarily characterized by older age (55–74 years). Comparing Wary and Unwary risk-takers directly (by re-setting the reference category, not shown in tables) underscored distinctive features of each. Compared to Wary Risk-takers, Unwary Risk-takers of both sexes were less likely to be from ethnic or sexual minorities, male Unwary Risk-takers were more likely to be aged 45–54 years, while female Unwary Risk-takers were more likely to be in a stable non-cohabiting relationship. The Highly Vulnerable class of women contained more sexual minorities than Unwary Risk-takers, and fewer ethnic minorities than Wary Risk-takers.

**Table 4:** Socio-demographic, health and lifestyle correlates of higher-risk latent sexual health classes (Men)

		Good Sexual Health	Sexual Function Problems		Wary Risk-takers		Unwary Risk-takers	
		(*n = 4112*, 83.1%	(*n = 458*, 8.8%)		(*n = 270*, 3.8%)		(*n = 273*, 4.3%)	
		%	%	Adjusted RRR	%	Adjusted RRR	%	Adjusted RRR
				(95% CI)		(95% CI)		(95% CI)
Stage (a) Sociodemographic factors								
Age	16–24	14.2	13.6	**0.59 (0.41–0.85)**	35.8	1.26 (0.85–1.87)	20.6	**0.64 (0.43–0.95)**
	25–44	40.8	48.4	1	40	1	36	1
	45–54	20.4	16.8	0.67 (0.43–1.03)	10.1	0.55 (0.26–1.17)	24.4	**1.65 (1.03–2.63)**
	55–74	24.6	21.3	0.78 (0.54–1.14)	14.1	0.89 (0.49–1.62)	19	1.29 (0.80–2.08)
Ethnicity	White	88.4	87.3	1	75.2	1	89.2	1
	Minority	11.6	12.7	1.31 (0.78–2.19)	24.8	**2.60 (1.63–4.12)**	10.8	0.94 (0.51–1.73)
Low SES score	0	50	51	1	27.9	1	37	1
	1 or 2	39	39.2	0.88 (0.65–1.20)	46	1.30 (0.82–2.06)	44.7	1.34 (0.93–1.95)
	3 to 5	11	9.8	0.74 (0.46–1.19)	26.1	**2.28 (1.33–3.91)**	18.3	**1.63 (1.00–2.65)**
Sexual identity	Heterosexual/straight	98.1	94.5	1	85.4	1	97.4	1
	Gay/bisexual/other	1.9	5.5	**3.65 (1.97–6.76)**	14.7	**9.23 (4.17–20.47)**	2.6	0.83 (0.24–2.83)
Partner	Married/cohabiting	75.8	69.1	1	31.7	1	42.2	1
	Not cohabiting	11.2	14.8	**1.76 (1.21–2.57)**	16.3	**2.83 (1.64–4.87)**	25.9	**5.18 (3.34–8.04)**
	No regular partner	13	16.1	**1.61 (1.14–2.28)**	52	**7.29 (4.72–11.27)**	32	**5.49 (3.62–8.32)**
Stage (b) Health and lifestyle factors								
Alcohol/drug abuse	no	78.9	72.7	1	54.7	1	59.4	1
	yes	21.1	27.3	**1.41 (1.03–1.92)**	45.3	**2.18 (1.50–3.16)**	40.6	**2.22 (1.59–3.11)**
Depression	no	93.2	79.5	1	86.8	1	86.7	1
	yes	6.8	20.5	**5.06 (3.41–7.51)**	13.2	**1.72 (1.04–2.85)**	13.3	**1.78 (1.10–2.87)**
Age at first sex	16+ years	73.6	73.6	1	56.9	1	50.5	1
	< 16 years	26.4	26.4	1.00 (0.74–1.35)	43.1	**1.73 (1.15–2.59)**	49.5	**2.59 (1.89–3.55)**

At stage (a) RRRs mutually adjusted for all other socio-demographic factors, stage (b) RRRs adjusted for socio-demographic factors in stage (a), but not mutually adjusted for other health/lifestyle factors. Bold font highlights statistically significant difference (*p* < .05) from the reference group. Percentages and RRRs allow for complex survey design features

*SES* socio-economic status (score of lowest household income quintile, no educational qualifications, unemployed; highest area deprivation quintile). *RRR* relative risk ratio, *CI* confidence intervals where Good Sexual Health is the reference group

Selected health and lifestyle correlates of latent sexual health class membership were explored next, adjusting each correlate for socio-demographic factors already considered (Tables 4 and 5, stage (b)). Depression was associated with membership of all higher-risk classes, among both men and women. Alcohol/drug use was associated with membership of all male poor sexual health classes, and all female classes reporting STI/unplanned pregnancy risk (including Highly Vulnerable women). Early sexual debut was associated with all male and female classes reporting STI/unplanned pregnancy risk, and with the female Low Interest class. Highly Vulnerable women were more likely to report early sexual debut than any of the other female poor sexual health classes.

**Table 5:** Socio-demographic, health and lifestyle correlates of higher-risk latent sexual health classes (Women)

		Good Sexual Health (n = 3675, 52.4%)	Wary Risk-takers (n = 143, 2.0%)		Unwary Risk-takers (n = 482, 6.9%)		Sexual Function Problems (n = 535, 7.6%)		Low Interest (n = 2046, 29.1%)		Highly Vulnerable (n = 138, 2.0%)	
		%	%	Adjusted RRR (95% CI)	%	Adjusted RRR (95% CI)	%	Adjusted RRR (95% CI)	%	Adjusted RRR (95% CI)	%	Adjusted RRR (95% CI)
Stage(a) socio-demographic factors												
Age	16–24	14	12.3	1.48 (0.90–2.44)	33.2	1.15 (0.82–1.63)	34.6	1.08 (0.74–1.56)	12.8	1.07 (0.77–1.49)	40	1.47 (0.89–2.41)
	25–44	44.2	42.2	1	39.8	1	46.2	1	40.4	1	41.4	1
	45–54	21.2	23.6	1.19 (0.59–2.39)	18.3	**0.53** **(0.31–0.93)**	12.6	1.22 (0.83–1.79)	20.9	1.18 (0.86–1.61)	11.8	0.69 (0.28–1.70)
	55–74	20.7	21.9	0.84 (0.30–2.36)	8.7	**0.33** **(0.15–0.76)**	6.6	1.23 (0.83–1.82)	25.9	**1.85** **(1.39–2.46)**	6.8	0.41 (0.11–1.55)
Ethnicity	White	88.5	90.1	1	66	1	87.9	1	89.5	1	86.6	1
	Minority	11.5	9.9	**3.78** **(2.28–6.25)**	34	0.84 (0.52–1.35)	12.1	0.95 (0.57–1.59)	10.5	1.09 (0.73–1.63)	13.5	1.40 (0.74–2.65)
Low SES score	0	44.5	48.8	1	24.1	1	25.4	1	44	1	26.3	1
	1 or 2	42.4	40.1	**1.83** **(1.07–3.15)**	52.8	**1.72** **(1.20–2.46)**	51	0.89 (0.66–1.20)	41.3	1.11 (0.86–1.43)	51	**2.05** **(1.14–3.69)**
	3 to 5	13.1	11.2	**2.18** **(1.11–4.26)**	23.1	**2.70** **(1.75–4.17)**	23.6	0.91 (0.59–1.40)	14.7	1.38 (0.97–1.97)	22.6	**2.31** **(1.15–4.64)**
Sexual identity	Hetero sexual/ straight	97.7	94.9	1	92.9	1	97.1	1	96.8	1	92.8	1
	Lesbian/ bisexual/ other	2.3	5.1	2.72 (0.98–7.55)	7.1	0.84 (0.37–1.89)	2.9	**2.46** **(1.20–5.06)**	3.2	1.65 (0.91–2.99)	7.2	3.24 (0.99–10.63)
Partner	Married/ Co- habiting	76.4	83.1	1	36.9	1	29.7	1	79.6	1	22.7	1
	Not co-habiting	12.6	9.2	**2.19** **(1.02–4.71)**	18.9	**6.09** **(3.79–9.79)**	28	**0.56** **(0.39–0.82)**	10.9	0.77 (0.54–1.09)	28.2	**6.87** **(2.79–16.92)**
	No regular partner	11	7.7	**6.05** **(3.41–10.72)**	44.2	**11.60** **(7.38–18.24)**	42.3	**0.51** **(0.31–0.85)**	9.4	0.75 (0.52–1.08)	49.2	**14.41** **(6.12–33.94)**
Stage (b) Health and lifestyle factors												
Alcohol/ drug use	no	84.7	81.7	1	69.4	1	69.2	1	82.2	1	55.1	1
	yes	15.3	18.3	**1.98** **(1.22–3.22)**	30.6	**1.74** **(1.24–2.45)**	30.8	1.36 (0.90–2.05)	17.9	1.34 (0.98–1.83)	44.9	**3.16** **(1.92–5.22)**
Depression	no	94.1	78	1	81.8	1	84.7	1	83.7	1	65.6	1
	yes	5.9	22	**4.01** **(1.88–8.56)**	18.2	**3.03** **(1.68–5.46)**	15.3	**11.03** **(6.55–18.59)**	16.4	**7.11** **(4.01–12.61)**	34.4	**11.76** **(6.37–21.73)**
Age at first sex	16+ years	82.7	78.6	1	73.2	1	63.8	1	79	1	48.1	1
	< 16 years	17.3	21.4	**1.78** **(1.12–2.83)**	26.8	**2.43** **(1.74–3.39)**	36.2	1.43 (.99–2.08)	21	**1.60** **(1.15–2.22)**	51.9	**5.43** **(3.31–8.92)**

At stage (a) RRRs mutually adjusted for all other socio-demographic factors, stage (b) RRRs adjusted for socio-demographic factors in stage (a), but not mutually adjusted for other health/lifestyle factors. Bold font highlights statistically significant difference (*p* < .05) from the reference group. Percentages and RRRs allow for complex survey design features

*SES* socio-economic status (score of lowest household income quintile, no educational qualifications, unemployed; highest area deprivation quintile). *RRR* relative risk ratio, *CI* confidence intervals where Good Sexual Health is the reference group

Lastly, we explored associations between latent sexual health class membership and respondents’ evaluations of their sex lives. Table 6 shows (for men and women) the percentage in each class with low satisfaction/high distress, together with the estimated probability of low satisfaction/high distress allowing for classification uncertainty. Among men, only the Sexual Function Problems class were more likely to perceive low satisfaction/high distress with their sex lives than those in Good Sexual Health. In contrast, all female poor sexual health classes were more likely to perceive low satisfaction/high distress with their sex lives. Among women, the highest probabilities of low satisfaction/high distress were found for the Sexual Function Problems, Low Interest and Highly Vulnerable classes.

**Table 6:** Associations between sexual health group and low satisfaction/high distress with sex life (Men and Women)

	Low satisfaction/high distress with sex life			
	%	95% CI	Probability (SE)	*p*-value for comparison with Good Sexual Health
Men				
Good Sexual Health	9.6	8.5 to 10.7	0.07 (0.01)	
Sexual Function Problems	31.6	27.0 to 36.6	0.40 (0.02)	<.001
Wary Risk-takers	9.0	5.9 to 13.5	0.10 (0.02)	0.257
Unwary Risk-takers	10.2	6.6 to 15.5	0.09 (0.02)	0.738
Women				
Good Sexual Health	4.5	3.8 to 5.4	0.02 (0.01)	
Sexual Function Problems	40.7	35.6 to 46.0	0.45 (0.04)	<.001
Unwary Risk-takers	8.6	6.0 to 12.2	0.05 (0.01)	0.009
Wary Risk-takers	10.7	6.5 to 17.1	0.12 (0.03)	0.003
Low Interest	18.4	16.1 to 20.9	0.21 (0.02)	<.001
Highly Vulnerable	32.7	24.8 to 41.7	0.32 (0.07)	<.001

Percentages allow for complex survey features. Estimated probabilities allow for classification uncertainty. Chi-squares *p*-values compare the probability of low satisfaction/high distress with sex life for each higher-risk class with the Good Sexual Health reference group

*CI* confidence interval, *SE* standard error

## Discussion

To our knowledge, this is the first study to explore clustering of adverse sexual health markers using nationally representative data. Among sexually-active members of the British general population, substantial minorities (17% of men, 47.5% of women) experienced poor sexual health, when compared to majority groups with low probability of any adverse outcome. Among men, poor sexual health classes were characterised as either risk-taking (two classes) or sexual function problems (one class). Among women, similar poor sexual health classes were identified, in addition to a large class (over a quarter of the sample) characterised by low interest in sex, and a small “Highly Vulnerable” class (2%) reporting a range of adverse experiences across all markers of sexual health. The different poor sexual health classes had distinctive socio-demographic profiles, but lifestyle and health factors (alcohol and drug use, depression and early sexual debut) were common across all poor sexual health classes. Among women (but not men), all poor sexual health classes had more negative evaluations of their sex lives than those in good sexual health.

We differentiated two classes exposed to HIV/STI and (women) unplanned pregnancy risk without functional problems, collectively comprising 9% of sexually active men and women. Unlike Wary Risk-takers, Unwary Risk-takers perceived themselves to be at low risk of HIV and other STIs, despite having condomless sex with new partners. Unwary Risk-takers constituted a sizeable proportion of all risk-takers (around half of male risk-takers, and three-quarters of female risk-takers). Our finding chimes with other studies finding that substantial proportions of men and women appear to underestimate STI risk [13–15]. Among men (but not women), differences in risk perceptions between Unwary and Wary Risk-takers were consistent with differences in self-reported STI diagnosis, and may reflect lower STI risk exposure for the Unwary males. Nonetheless, Unwary Risk-takers’ high levels of unprotected sex give cause for concern, especially as low risk perception may be a barrier to condom use and STI testing [49]. Compared to Wary Risk-takers, Unwary classes were more likely to be heterosexual, in midlife (men) or in a steady non-cohabiting relationship (women). Risk awareness and STI/HIV testing may be difficult to promote among these comparatively low-risk groups, who may have competing priorities for intimacy, expectations for health and social norms on health-seeking [50, 51].

Among women, the combined size of classes reporting sexual function difficulties but low STI/HIV or unplanned pregnancy exposure (38%) considerably exceeded the corresponding male Sexual Function Problems class (9%). The greater prevalence of sexual function problems among women has been noted by others [2]. Our study underscores the known greater diversity of women’s sexual responses over men’s [52], which we found to be dominated by a large class expressing a lack of interest in sex. Low desire has been identified as the most common female sexual problem across many studies [2, 52–54]; and has been linked to relational factors including lower emotional closeness and difficulty communicating about sex [55]. In our study this class was not simply delineated by post-menopausal age, although in limiting our sample to those reporting sex within the past year we may have underestimated the effect of age and the size of this class in the wider population. Despite reporting relatively few physiological problems related to sexual functioning, these women’s more negative evaluations of their sex lives suggests practitioners should not overlook this group, and the likely detrimental impact of a less satisfying sex life on overall subjective wellbeing [56, 57].

In addition to the two larger female classes expressing sexual function problems, a small “Highly Vulnerable” class had elevated risk of functional problems accompanied by risk of STIs, unplanned pregnancy, and sexual coercion. Others have found associations between low sexual function and these other adverse outcomes, for men, as well as women [2, 6, 7, 58]. Our study did not find a corresponding male Highly Vulnerable class: this might reflect lower statistical power among the smaller male sample, together with lower male reporting of non-volitional sex. Particular concern arises from the pattern of risk markers seen in the Highly Vulnerable women, where low perceptions of risk seem inaccurate given their high levels of condomless sex, accompanied by the highest probability of STI diagnosis across all female classes. Our study highlights the potential significance of sexual coercion as a unifying attribute of sexual risk-taking and functional problems among women, since exposure to a broad spectrum of negative sexual experiences is likely to reflect women’s agency due to partner imbalances in sexual intent, communication and control [59, 60].

Despite the greater diversity of women’s sexual health compared to men’s that we have found, there were clear associations between all aspects of poor sexual health and low satisfaction/high distress with one’s sex life only among women. For men, only the class with sexual function problems had low satisfaction/high distress with their sex lives, consistent with other research on the impact of impaired sexual functioning on men’s satisfaction and distress [61]. The links we found between women’s membership of higher-risk classes and negative evaluation of their sex lives in part reflect the impact of sexual function problems or (for the highly vulnerable women) coercive sex on women’s sexual well-being found elsewhere [22, 62]. Associations found between exposure to STI or unplanned pregnancy risk and low satisfaction/high distress found among women only might reflect a greater dependency on relationship factors for both risk avoidance and sexual wellbeing [63, 64], as well as greater risk aversion [65].

Limitations of the study include the survey response rate, although this is in line with other national surveys undertaken at the time [66, 67]; and reliance on self-reported data. Although Natsal-3 included biosampling to test for a range of STIs [9], this was only available for a random sub-sample aged 16–44 years, preventing the inclusion of these data in our latent class analysis of respondents from across Natsal-3’s age-range 16–74 years. Risks of bias were mitigated via use of survey weights and self-completion modules for sensitive questions. We were limited to items included in the Natsal-3 survey, which was not specifically designed for this analysis. In the interests of providing a comprehensive range of measures, we included some with a relatively long look-back period that may limit their reliability as markers of current sexual health. Markers of non-volitional sex did not encompass the full range of behaviours indicative of coercive sexual relationships or establish severity [68, 69], and we did not include severity in our markers of sexual function. In order to include those not in longer-term sexual relationships (and avoid imputing such data), we omitted Natsal items on the quality of partner relationships used in other studies of sexual function [70, 71]. Our examination of sexual health class correlates focused on selected socio-demographic, lifestyle and health factors identified as important in previous analyses of individual outcomes [2, 6, 9, 10]. This does not represent an exhaustive list of information contained in Natsal-3, and additional potential correlates such as partner history and sex education could be investigated in a further study. The data are cross-sectional, and we cannot assume that associations described reflect causal effects. Causal mechanisms underlying associations between depression, substance use and sexual health are likely to be complex and bidirectional [72–74].

## Conclusions

A greater prevalence and diversity of poor sexual health appears to exist among women than men in Britain, with more consistent effects on women’s satisfaction/distress with their sex lives. Among both men and women, our novel population categories point to the existence of several important risk classes in danger of being overlooked by sexual health intervention efforts. Specifically these are male and female Unwary Risk-takers, female Low Interest and female Highly Vulnerable classes. Their profiles differed from those traditionally considered at risk of sexual dysfunction or STIs, suggesting the need for tailored interventions. Distinctive socio-demographic profiles should assist identification and targeting of these groups.

A further contribution of this study is also in highlighting *shared,* as well as distinctive, characteristics of poor sexual health groups. Depression, alcohol/drug use and young age of sexual debut were widely associated with membership of higher-risk classes. Of particular concern are the greater risks of early sexual debut among the Highly Vulnerable group of women, accompanied by depression and substance abuse. These factors have been widely implicated in studies of sexual risk-taking, functional problems or non-volitional sex [2, 6, 11, 12, 75–77]. Our study provides a clear demonstration that mutually exclusive clusters of problems (sexual risk-taking without functional problems; and the opposite) nonetheless have common underlying attributes, strengthening the notion of sexual health as a unifying “umbrella” concept that needs to be viewed – and treated - holistically. At a time when financial pressures are being felt by sexual health services across Britain [78], it may be advisable to prioritise upstream interventions with the most widespread (and cost-effective) benefits. Our findings reinforce existing evidence that age of sexual debut, substance use and comorbid depression are important targets for policy makers and practitioners concerned with improving sexual health at an individual and population level, pointing to their potential value in mitigating a broad spectrum of sexual health problems.

## Data Availability

The dataset supporting the conclusions of this article is available in the UK Data Service repository, unique persistent identifier: 10.5255/UKDA-SN-7799-1; https://beta.ukdataservice.ac.uk/datacatalogue/studies/study?id=7799&type=Data%20%20catalogue.

## Abbreviations

AIC: Akaike Information Criterion
BIC: Bayesian Information Criterion
BLRT: Bootstrap Likelihood Ratio Test
CI: confidence interval
FIML: Full Information Maximum Likelihood
HIV: human immunodeficiency virus
LMR: Lo, Mendell and Rubin Likelihood Ratio Test
Natsal: National Survey of Sexual Attitudes and Lifestyles
*p*: probability
RRR: relative risk ratio
SE: standard error
SES: socio-economic status
STI: sexually transmitted infection
WHO: World Health Organization
